# Sarcoidosis Presenting as Acute Respiratory Distress Syndrome

**DOI:** 10.1155/2018/6465180

**Published:** 2018-12-23

**Authors:** Bilal Haider Lashari, Ahmad Raza, Vincent Chan, William Ward

**Affiliations:** ^1^Department of Internal Medicine, Abington Hospital–Jefferson Health, Abington, PA, USA; ^2^Department of Pulmonary and Critical Care Medicine–Jefferson Health, Abington, PA, USA

## Abstract

Sarcoidosis is a multisystem granulomatous disease of unknown origin. It typically involves the lungs and mediastinal lymph nodes in a chronic fashion. However, acute syndrome has been reported possibly in response to systemic release of proinflammatory cytokines. Acute pulmonary manifestations, especially acute respiratory failure or acute respiratory distress syndrome, remain extremely uncommon in individuals without a prior diagnosis. We present the case of a 41-year-old African American female, who presented with ARDS. An extensive workup into the cause of her illness remained negative, and she subsequently succumbed to her illness. A diagnosis of sarcoidosis was made upon autopsy, after exclusion of other granulomatous illness. The case highlights the need to consider this uncommon diagnosis in patients with unexplained ARDS to guide therapy.

## 1. Introduction

**Figure 1:**
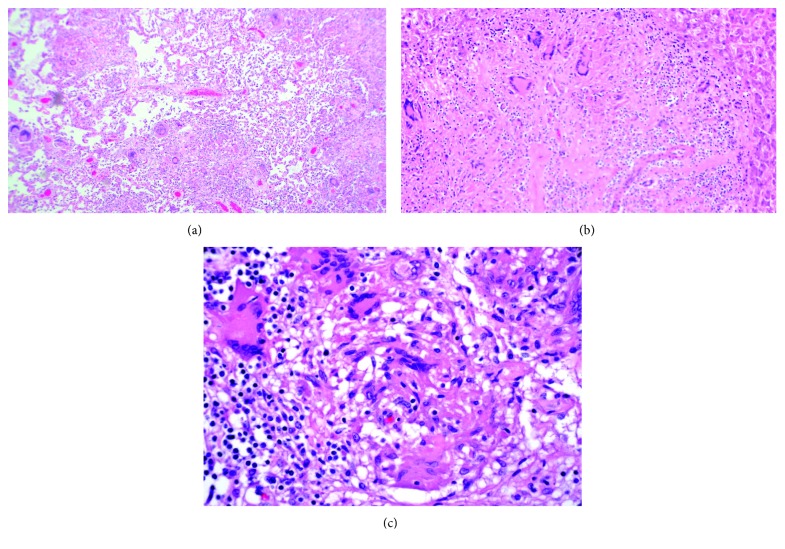
Histopathology. (a) Low-power view of lung parenchyma showing multiple giant cell formation and granuloma formation. (b) Giant cell and granuloma formation in hepatic parenchyma. (c) High-power view of granulomas and giant cells in the lymph node.

**Figure 2:**
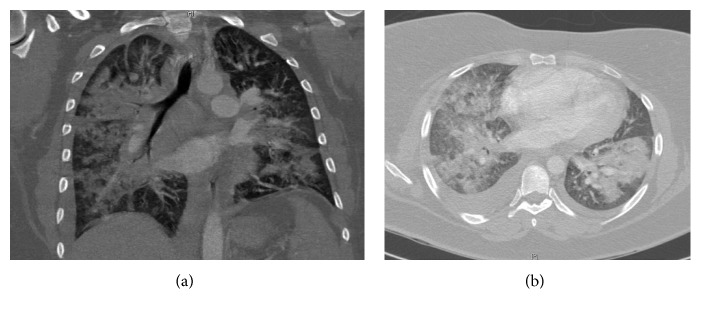
Radiographic appearance at presentation.

Sarcoidosis is a multisystem granulomatous disease of unknown etiology. Frequently affecting the mediastinal lymph nodes and lungs, cutaneous, hepatic, and ocular manifestations are also common. It can also involve the myocardium, central nervous system, spleen, and upper respiratory tract. Involvement is primarily in the form of nonnecrotizing granuloma formation, in response to T-cell-mediated delayed hypersensitivity reaction, recruiting macrophages and release of inflammatory cytokines TNF-a and IL-2. Pulmonary involvement is usually slow, leading to a chronic interstitial lung disease with progressive dyspnea, cough, and fatigue ailing affected individuals. Acute pulmonary presentations are uncommon and typically occur in advanced sarcoidosis in response to infection, bronchospasm, or injury causing deterioration of the respiratory status. Even less common is an acute initial presentation with respiratory failure in a previously undiagnosed patient, with only a handful of cases reported in the English literature. We describe one such case of a young woman with acute respiratory distress syndrome and respiratory failure diagnosed as sarcoidosis posthumously (Figures 1 and 2).

## 2. Case

A 41-year-old African American female presented to the emergency department with a cough, dyspnea, fevers, chills, night sweats, and fatigue.

She had never experienced pulmonary symptoms two weeks before presentation when she developed a cough and fever and was prescribed oral levofloxacin for pneumonia by her primary care physician. Completing a 7-day course of antibiotics, with unabating symptoms and worsening dyspnea, she presented to the emergency department for further treatment.

The examination was remarkable for tachypnea with a respiratory rate of 30/min, hypoxemia with an oxygen saturation of 87% in room air, and diffuse bilateral crackles, without jugular venous distension, or lower extremity edema.

Blood test was significant for white blood cell count of 30,000/*µ*L, lactic acid of 5 mEq/L, and a normal metabolic panel. Arterial blood gas revealed a pH of 7.17, PaCO_2_ of 50 mmHg, HCO_3_ of 19 mmol/L, PaO_2_ of 65.3 mmHg, and SaO_2_ of 87%.

A CT-PE of the chest showed bilateral extensive multifocal infiltrates with significant hilar and mediastinal lymphadenopathy and no evidence of pulmonary embolism.

She was intubated for respiratory distress and admitted to the medical intensive care unit, with a tentative diagnosis of sepsis secondary to pneumonia, and started on broad-spectrum antibiotics.

A parasite smear and initial blood cultures were negative.

Bronchoscopy done on the day of admission showed mild diffuse erythema without hemorrhage. Given the patient's repeated desaturation during the procedure, transbronchial biopsies were not performed and the procedure terminated early. Lavage was sent for cytology, bacterial, mycobacterial, and fungal stain, and culture.

An echocardiogram showed a hyperdynamic left ventricle with an estimated ejection fraction of 70% with severe right ventricular dilatation and hypokinesis. Right ventricular systolic pressure was estimated at 80 mmHg with a tricuspid annular plane systolic excursion (TAPSE) of 1.3.

She continued to worsen, with worsening hypoxia and difficulty with mechanical ventilation, ultimately requiring airway pressure release ventilation and inhaled epoprostenol. Bronchoalveolar lavage remained negative, for bacteria, fungi, or *Pneumocystis jirovecii* sp.

Workup was expanded to include fungal blood cultures, histoplasma and coccidioides serology, and viral respiratory panel. Respiratory syncytial virus, adenovirus, influenza, parainfluenza, human metapneumovirus, human immunodeficiency virus (HIV), Ebstein–Barr virus (EBV), and cytomegalovirus (CMV) were negative. Leptospira and pertussis remained negative. Noninfectious workup along autoimmune lines including antinuclear antibody (ANA), anti-neutrophilic cytoplasmic antibody (ANCA), rheumatoid factor (RF), anti-cyclic citrullinated peptide (anti-CCP), and anti-Smith, anti-SCL70, anti-myeloperoxidase (anti-MPO), anti-ribonucleoprotein (anti-RNP), and anti-PR3 antibodies all returned negative, with angiotensin-converting enzyme (ACE) levels on the high end of normal.

She continued to worsen, requiring hemodynamic support with norepinephrine, epinephrine, and vasopressin, with worsening hepatic function and oliguric renal failure.

On the third hospital day, she continued to be febrile. Morning ABG showed a pH of 6.99, PaCO_2_ of 47.4 mmHg, HCO_3_ of 10 mmol/L, PaO_2_ of 88.2 mmHg, SO_2_ of 83.2%, and lactic acid level of 9 mEq/L; after prompt discussion with the family, the decision was made to start the patient on continuous renal replacement therapy (CRRT) and consider extracorporeal membrane oxygenation (ECMO) support.

However, the patient developed pulseless electrical activity (PEA) arrest before CRRT could begin, with the eventual demise of the patient.

An autopsy revealed noncaseating granulomata in hilar and mediastinal lymph nodes and pulmonary, splenic, and hepatic parenchyma. Gram, Ziehl–Neelsen, and special stains were negative for bacterial, mycobacterial, fungal, or parasitic organisms.

Acid-fast bacillary and fungal cultures remained negative. No crystals were seen on polarized microscopy.

## 3. Discussion

Overall, African Americans have a threefold age-adjusted annual incidence of sarcoidosis when compared to Caucasians [1].

There is no diagnostic laboratory or imaging study available. A joint consensus statement recommends three criteria for the diagnosis of sarcoidosis: (a) a compatible clinical and radiologic presentation, (b) pathologic evidence of noncaseating granulomas, and (c) exclusion of other diseases with similar findings [2]. Clinical suspicion is critical for diagnosing sarcoidosis, a constellation of consistent symptoms, and supporting imaging is of paramount importance.

Although the initial diagnosis is made on clinicoradiographic findings, a tissue biopsy is warranted to confirm the diagnosis. The presence of noncaseating granulomas demonstrated on microscopic evaluation is typical. However, other granulomatous disorders need to be excluded before a determination can be made. The diagnosis is made even more difficult when clinical suspicion is low, owing to different presentations like in our case.

Our patient met the Berlin criteria for ARDS at presentation. Owing to the lack of a satisfactory underlying diagnosis, an extensive workup [3] was performed which remained negative. She succumbed to her illness, primarily from worsening ARDS and right heart failure. The demonstration of granulomata in the mediastinal nodes, pulmonary parenchyma, and hepatic tissue suggested the diagnosis posthumously, which was made after staining and culture for microbial pathogens, including mycobacteria and fungi.

Sarcoidosis does not generally cause a fulminant pulmonary syndrome, as in our patient. Only scattered case reports exist of febrile illness attributed to sarcoidosis outside of the well-described Heerfordt-Waldenström syndrome, also referred to as sarcoid uveoparotid fever [4].

An acute manifestation of sarcoidosis most commonly presents as Löfgren's syndrome, characterized by the classic triad of hilar adenopathy, erythema nodosum, and arthritis.

Lung involvement is most common in sarcoidosis. However, acute respiratory failure and acute respiratory distress syndrome are incredibly rare initial presentations of sarcoidosis, with a handful of reports in the literature [5–12].  Table 1 provides some details about reported cases.

Interestingly, acute febrile presentations appear to be extremely rare. Suyuma et al. have previously described a similar patient with high fevers and acute respiratory failure diagnosed subsequently as pulmonary sarcoidosis [12].

Previously, authors have termed a rapidly manifesting variant as alveolar sarcoidosis, owing to the acuity of onset and radiologic pattern consistent with diffuse alveolar infiltrates and presence of air bronchograms. Interestingly, most patients diagnosed with alveolar sarcoidosis also did not present with respiratory failure. Two such cases have been documented, by Sahn et al. [11] and Gera et al. [6].

An acute inflammatory syndrome is poorly understood and postulated in response to the systemic activation of T-cell-mediated immune response and activation of the interferon-gamma and interleukin-2 pathways which can then lead to the development of ARDS [8].

Sarcoidosis is not a usual cause of ARDS and is seldom cited as the underlying pathology in these patients; as our case and other cases before illustrated, it is essential to consider this in the differential for unexplained ARDS.

This is the only reported case of ARDS from an acute febrile presentation of sarcoidosis which was diagnosed posthumously, highlighting the importance of considering it in patients with ARDS from an unknown underlying disease process.

## 4. Conclusion

Acute respiratory failure due to ARDS is an underrecognized presentation of sarcoidosis and can be potentially fatal if not treated promptly. Physicians need to be vigilant in considering sarcoidosis as a cause of ARDS after excluding common etiologies.

## Figures and Tables

**Table 1:** 

Author	Year	Patient	Symptoms	Duration of symptoms	Radiographic findings	Biopsy	Treatment and outcome
Sahn et. al. [11]	1974	26, female	Dyspnea, productive cough, and chest pain	1 month	Bilateral diffuse infiltrates without adenopathy	Open lung biopsy	Improvement with prednisone
Suyama et. al. [12]	1990	55, male	Dyspnea and fever	10 days	Diffuse small nodular opacity, with bilateral lymphadenopathy	Transbronchial lung biopsy	Improvement with prednisone
Sabbagh et al. [9]	2002	50, male	Dyspnea and productive cough	3 weeks	Extensive bilateral interstitial infiltrates	Transbronchial lung biopsy	Improvement with prednisone
Leiba et al. [13]	2004	41, female	Dyspnea	1 week	Bilateral reticulonodular opacities	Mediastinal lymph node biopsy	Improvement with prednisone
Chirakalwasan and Dallal [14]	2005	33, male	Acute respiratory distress syndrome	4 days	Bilateral pulmonary infiltrates	Transbronchial lung biopsy	Improvement with methylprednisolone
Shibata et al. [10]	2007	66, male	Leg rash, fever, and acute respiratory failure	n/a	Bilateral ground glass opacity	Skin biopsy	Improvement with steroids
Gupta et al. [7]	2011	40, male	Dyspnea, cough, fever, malaise, and weight loss	3 weeks	Bilateral patchy ground glass opacity with interlobular thickening and scattered nodules	Lung biopsy	Improvement with methylprednisolone
Gera et al. [6]	2014	30, male	Dyspnea, cough, and fever	20 days	Bilateral lymphadenopathy and confluent alveolar opacities with air bronchograms	Transbronchial biopsy	Improvement with prednisone
Arondi et al. [5]	2016	20, female	Dry cough, fatigue, and fever	1 week	Bilateral lymphadenopathy with diffuse ground glass opacities	Transbronchial biopsy	Improvement with methylprednisolone

## References

[1] Rybicki B. A., Major M., Popovich J., Maliank M. J., lannuzzi M. C. (1997). Racial differences in sarcoidosis incidence: a 5-year study in a health maintenance organization. *American Journal of Epidemiology*.

[2] AJRCC, s (1999). Statement on sarcoidosis. Joint statement of the American thoracic society (ATS), the European respiratory society (ERS) and the world association of sarcoidosis and other granulomatous disorders (WASOG) adopted by the ATS board of directors and by the ERS executive committee. *American Journal of Respiratory and Critical Care Medicine*.

[3] Papazian L., Calfee C. S., Chiumello D. (2016). Diagnostic workup for ARDS patients. *Intensive Care Medicine*.

[4] Denny M. C., Fotino A. D. (2013). The Heerfordt-Waldenstrom syndrome as an initial presentation of sarcoidosis. *Baylor University Medical Center Proceedings*.

[5] Arondi S., Valsecchi A., Borghesi A., Monti S. (2016). Pulmonary sarcoidosis presenting with acute respiratory distress syndrome. *Annals of Thoracic Medicine*.

[6] Gera K., Gupta N., Ahuja A., Shah A. (2014). Acute alveolar sarcoidosis presenting with hypoxaemic respiratory failure. *BMJ Case Reports*.

[7] Gupta D., Agarwal R., Paul A. S., Joshi K. (2011). Acute hypoxemic respiratory failure in sarcoidosis: a case report and literature review. *Respiratory Care*.

[8] Pugazhenthi M. (2005). Sarcoidosis presenting as acute respiratory failure. *Southern Medical Journal*.

[9] Sabbagh F., Gibbs C., Efferen L. S. (2002). Pulmonary sarcoidosis and the acute respiratory distress syndrome (ARDS). *Thorax*.

[10] Shibata S., Saito K., Ishiwata N., Ieki R. (2007). A case of sarcoidosis presenting with high fever and rash progressing to acute respiratory failure. *Nihon Kokyuki Gakkai Zasshi*.

[11] Sahn S. A., Schwarz M. I., Lakshminarayan S. (1974). Sarcoidosis: the significance of an acinar pattern on chest roentgenogram. *Chest*.

[12] Suyama T., Satoh H., Inoue T. (1990). A case of sarcoidosis presenting with high fever and acute respiratory failure. *Kekkaku*.

[13] Leiba A., Apter S., Leiba M., Thaler M., Grossman E. (2004). Acute respiratory failure in a patient with sarcoidosis and immunodeficiency? An unusual presentation and a complicated course. *Lung*.

[14] Chirakalwasan N., Dallal M. M. (2005). Pulmonary sarcoidosis presenting with acute respiratory failure. *Southern Medical Journal*.

